# Comparison of HBV-specific T cell reactivity across the pregnant, postpartum and non-pregnant women with chronic HBV infection

**DOI:** 10.3389/fimmu.2024.1461767

**Published:** 2024-10-10

**Authors:** Genju Wang, Fangping Yue, Ziyue Zhang, Yandan Wu, Ruixue Ji, Guanlun Zhou, Ying Ji, Chuanlai Shen

**Affiliations:** ^1^ Department of Obstetrics and Gynecology, The Second Hospital of Nanjing, Affiliated to Nanjing University of Chinese Medicine, Nanjing, Jiangsu, China; ^2^ Department of Microbiology and Immunology, Medical School of Southeast University, Nanjing, Jiangsu, China

**Keywords:** chronic hepatitis B infection, pregnancy, antigen-specific T cell detection, ELISpot assay, postpartum women

## Abstract

**Objective:**

To investigate the features of HBV-specific T cell reactivity across the pregnant, postpartum or non-pregnant women with chronic HBV infection.

**Methods:**

A total of 283 patients with chronic HBV infection were enrolled in this study, including 129 patients during pregnancy, 58 patients during postpartum less than 6 months and 96 non-pregnant patients at childbearing age. A universal ELISpot assay was set up using a broad-spectrum T-cell epitope peptide library which containing 103 functionally validated CD8^+^ T-cell epitopes derived from overall HBsAg, HBc/eAg, HBx and HBpol proteins and fitting to the human leukocyte antigen polymorphisms of Chinese population. Then, The functional HBV-specific T cells in peripheral blood were detected.

**Results:**

The spot-forming units (SFUs) of HBV-specific T cells in the pregnant group showed no statistical difference from the postpartum group, but significantly less than that in the non-pregnant group (p = 0.046). In the untreated patients, the pregnant group displayed HBe/cAg-specific T cells (SFUs) less than the non-pregnant group (*P* = 0.025) and the postpartum group (*P* = 0.045). Meanwhile, in the NUCs-treated patients, the three groups presented similar HBV-specific T cell reactivity. Furthermore, the SFUs in the NUCs-treated pregnant group were similar to that in the NUCs-untreated pregnant group. Importantly, ROC analysis demonstrated that the HBV-specific T cells (SFUs) (AUC = 0.742) and combined with HBsAg levels (AUC = 0.775) or with HBeAg level (AUC = 0.78) had a good predictive performance for hepatitis progression during pregnancy group.

**Conclusion:**

Pregnancy can reduce HBV-specific T cell reactivity in the women with chronic HBV infection, and NUCs treatment cannot improve their HBV-specific T cells reactivity. Routine monitoring of HBV-specific T cells during pregnant and postpartum period can provide precise evaluation for immune function and valuable guidance for treatments.

## Introduction

Hepatitis B virus (HBV) vaccines have been available for more than 30 years, but the infection has still spread around the world. About 10% of the world’s population is infected with HBV, and more than 257 million of whom suffer from chronic lifelong infection (1, 2). China has the largest burden of HBV infection in the world, with about 70 million people currently infected, and 20-30 million patients with chronic hepatitis B (3). More importantly, maternal-neonatal route is the main way of HBV transmission currently (4). Approximately 75 million women at childbearing age are under chronic HBV infection worldwide (5). For the pregnant woman with chronic HBV infection, the immunity presents special features. For example, the levels of steroid hormones such as estrogen, cortisol and aldosterone are significantly increased, which is one of the reasons for the inhibition of T cell immune function; The success of pregnancy is accompanied by the suppression of immune rejection and the establishment of maternal immune tolerance; Most pregnant women with chronic HBV infection are in the immune tolerance phase, that means high viral load, normal liver function and no clinical symptoms. Around 6-14% of pregnant women progress to hepatitis during pregnancy, and 3.5-50% women progress to hepatitis after delivery, but the immunological mechanism remains unclear (6).

HBV-specific T cell plays a very important role in antiviral infection and is an important factor affecting the disease course and outcome, among which HBV-specific CD8^+^ T cell-mediated cytotoxic effects play a critical role in clearing the virus. In the patients with chronic hepatitis B (CHB), the specific T cells are mainly manifested by functional exhaustion, such as increased expression of PD-1 and CTLA-4, increased IL-10 secretion and decreased IFN-γ and IL-2 production, or increased Treg cells (7–9). Previous studies have mostly been conducted for the non-pregnant patients. Little is known on HBV-specific T cells in pregnant women with chronic HBV infection. Whether short-term antiviral treatment during pregnancy affects the disease progression in immune-tolerant mothers remains controversial. Clarifying the functional characteristics of HBV-specific T cells during pregnancy will be useful to guide the antiviral therapy and postpartum treatments.

## Patients and methods

### Patient cohort

A total of 283 patients with chronic HBV infection were enrolled in this study from the Division of Obstetrics and Gynecology at Nanjing Second Hospital, including 129 ones during pregnancy, 58 ones during postpartum less than 6 months and 96 non-pregnant ones at childbearing age. The inclusion criteria are as follows: 1) Female patients aged 20-39 years; 2) Patients who have been HBsAg positive for more than 6 months; 3) Have clinical, biochemical and viral symptoms of chronic HBV infection. The exclusion criteria are as follows: 1) Patients involved in hepatitis A, hepatitis C, human immunodeficiency virus or other viral infections; 2) Those with other chronic liver diseases (autoimmune liver disease, alcoholic liver disease, fatty liver, etc.); 3) Patients with cirrhosis, liver cancer or obstetric diseases (such as intrahepatic cholestasis during pregnancy). The clinical baseline features and nucleos(t)ide analogue (NUC) treatment regimens of these patients are presented in **Table 1**. All participants provided written informed consent and the Ethics committee approval conforming to the Declaration of Helsinki was obtained from Clinical Ethics Committee of Nanjing Second Hospital (ref: 2024-LS-ky041).

**Table 1:** Baseline features about the research subjects.

	Pregnant group Median (min-max)	Postpartum group Median (min-max)	Non-pregnant group Median (min-max)	K-W/*P*	M-W/*P*
Total patients	129	58	96		
Patients with TDF treatment for 3-6 months (300mg QD)	17	26	63		
Age (years)	31 (25 - 41)	32 (22 - 42)	33 (22 - 59)	0.227	Preg vs Post 0.227 Preg vs Non-p 0.356 Post vs Non-p 0.148
HBV-specific T cells (SFUs/4×10^5^ PBMCs)	79.0 (5 - 489)	77.5 (14 - 652)	91.0 (12 - 949)	0.149	Preg vs Post 0.358 Preg vs Non-p **0.046** Post vs Non-p 0.259
ALT (IU/L)	18.5 (6.5 - 160.2)	24.9 (9.9 - 178.2)	23.1 (7.1 - 136.8)	0.256	Preg vs Post 0.159 Preg vs Non-p 0.487 Post vs Non-p 0.301
AST (IU/L)	19.3 (10 - 65)	22.6 (12.6 - 162.2)	23.1 (12.9 - 143)	0.318	Preg vs Post 0.207 Preg vs Non-p 0.195 Post vs Non-p 0.513
HBV DNA (lg IU/mL)	2.92 (0.30 - 8.87)	1.83 (0.30 - 8.23)	0.88 (0.30 - 8.62)	0.09	Preg vs Post 0.123 Preg vs Non-p 0.08 Post vs Non-p 0.102
HBsAg (IU/mL)	5762 (0.68 - >52000)	10560.5 (< 2 - > 52000)	2211 (< 0.05 - > 52000)	0.104	Preg vs Post 0.07 Preg vs Non-p 0.137 Post vs Non-p 0.109
HBsAb (IU/mL)	< 2 (< 2 - 14.9)	< 2 (< 2 - 454.5)	< 2 (0.05 - 18.54)	0.903	Preg vs Post 0.859 Preg vs Non-p 0.926 Post vs Non-p 0.897
HBeAg (COI)	1.760 (0.009 - 7878.8)	6.445 (0.07 - 1732)	0.354 (0.076 - 1644)	0.19	Preg vs Post 0.148 Preg vs Non-p 0.251 Post vs Non-p 0.165
HBeAb (COI)	1.000 (0.002 - 8.620)	1.100 (0.002 - 6.620)	0.412 (0.002 - 7.180)	0.761	Preg vs Post 0.865 Preg vs Non-p 0.391 Post vs Non-p 0.416
HBcAb (COI)	0.009 (0.006 - 0.091)	0.009 (0.006 - 26.300)	0.009 (0.006 - 8.140)	0.904	Preg vs Post 0.853 Preg vs Non-p 0.879 Post vs Non-p 0.924

ALT (reference values: <40 IU/L), HBV-DNA (reference values: <2.69 lg IU/mL), HBsAg (reference values: 0-0.05 IU/mL), HBsAb (reference values: 0-10 IU/mL), HBeAg (reference values: 0-1 COI), HBeAb (reference values: 1-99 COI), HBcAb (reference values: 1-99 COI). COI, cut off index, COI = sample value/cut off value.

### Broad-spectrum HBV CD8^+^ T-cell epitope peptide library

The CD8^+^ T-cell epitopes validated in house were incorporated with the CD8^+^ T-cell epitopes functionally defined by other researchers to establish a HBV-specific antigenic peptide library which contained 103 epitopes. As confirmed by several methods, these epitope peptides were cross-presented by 13 predominant human leucocyte antigen (HLA)-A allotypes (A1101, A2402, A0201, A0207, A3303, A0206, A3001, A0203, A3101, A1102, A0101, A2601, A0301) which gather a total gene frequency of around 95% in Chinese and Northeast Asian populations (10). Then, these validated epitope peptides were grouped into eight peptide pools according to their derived proteins (HBsAg, HBpol, HBx and HBe/cAg), and each protein included two peptide pools. Notably, the 25 epitope peptides in peptide pool 7 and pool 8 were screened and verified from the whole sequence of HBeAg (including the HBcAg sequence) (**Supplementary Table S1**). Finally, the IFN-γ ELISpot assay was established using the peptide pool array to routinely enumerate the reactive HBV-specific T cells in peripheral blood of HBV-infected patients (11).

### ELISpot assay and detection of HBV-specific T cells

Peripheral blood mononuclear cells (PBMCs) were routinely prepared and seeded into 10 wells (4×10^5^ cells/well) in the 96-well ELISpot plates which were pre-coated with human IFN-γ ELISpot capture antibody (BD Biosciences, 1:200 dilution), and cocultured with eight peptide pools for 20 hrs in 5% CO_2_ incubator at 37°C (one well/peptide pool, 2 μg/peptide/well, totally eight experimental wells). In parallel, negative control well (PBMCs alone) and positive control well (2× 10^5^ PBMCs with phytohemagglutinin, PHA, 2.5 mg/well) were also performed. Notably, in each negative control well, DMSO was supplemented to make its concentration equal to the peptide pool/PBMCs co-culture wells. Then, spots were developed with human IFN-γ detection antibody (BD Pharmingen, 1:250 dilution), streptavidin-HRP (BD Pharmingen, 1:100 dilution) and AEC substrate set (BD Pharmingen,1:50 dilution), according to manufacturer’s protocols. The spot forming units (SFUs) were imaged and enumerated. SFUs in each experimental well = actual spot count in each experimental well - actual spot count in negative control well. When the actual spot count in the indicated experimental well is less than that in the negative control well, SFUs in the indicated experimental well is taken as 0 SFUs. Finally, the total SFUs for eight peptide pools is the sum of SFUs/4×10^5^ PBMCs in eight experimental wells. Meanwhile, the SFUs/4×10^5^ PBMCs for each indicated HBV protein were also calculated according to the peptide pools derived from each protein.

### Sero-virological parameters detection

The levels of HBsAg, HBeAg, HBsAb, HBeAb, HBcAb, ALT and AST were quantified using Roche Cobas 8000 Detection System. The HBV DNA load was detected by quantitative real-time PCR and Roche kits. These real-time data from the clinical laboratory of Nanjing Second Hospital were collected at the time point of HBV-specific T cells test for each patient.

### Statistical analysis

Statistical analysis was performed using GraphPad Prism 9 (GraphPad, La Jolla, CA, USA). Data were presented as median (interquartile range). A Mann-Whitney (non-parametric) test was used for the analyses of HBV DNA and ALT, SFUs, HBsAg and HBeAg medians between two groups. Kruskal-Wallis test (non-parametric) was performed when analyzing more than two groups. The predictive efficacy of HBV-specific T cells and sero-virological parameters on hepatitis progression in pregnant patients with chronic HBV infection was assessed by receiver operating characteristics (ROC) and area under the ROC curve (AUC). The DeLong test was performed for the comparison of different ROC curves. P < 0.05 was considered statistically significant.

## Results

### Comparison of HBV-specific T cell reactivity across the pregnant, postpartum and non-pregnant groups

**Figure 1:**
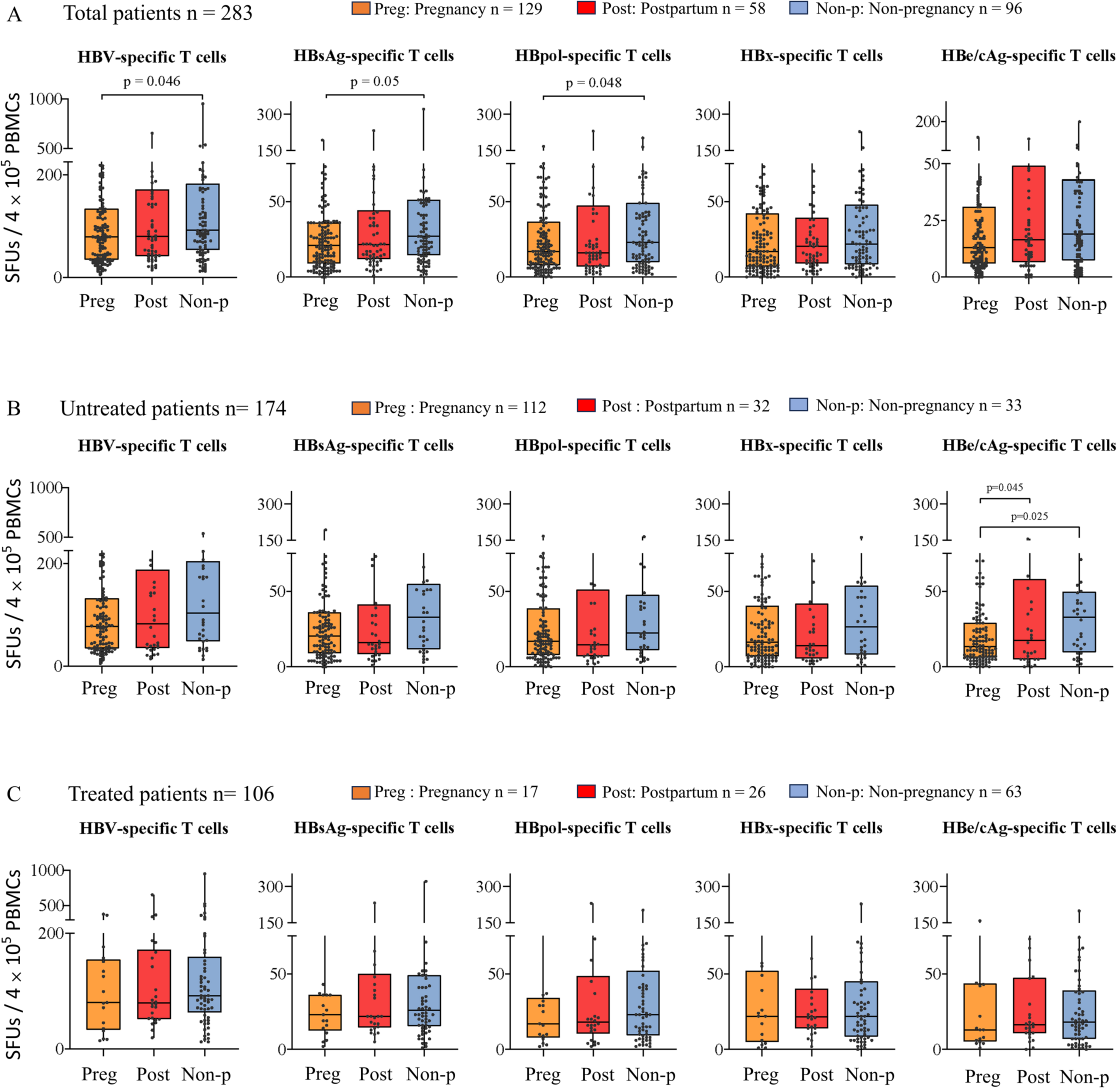
Comparison of HBV-specific T cell reactivity across the pregnant, postpartum and non-pregnant patients with chronic HBV infection. Reactive HBV-specific T cells in PBMCs were detected using ex vivo IFN-γ ELISpot assay and 103 validated T-cell epitope peptides. The total HBV-specific T cells and the indicated antigen-specific T cells (SFUs) in the pregnant, postpartum and non-pregnant groups were presented for 283 patients with chronic HBV infection **(A)**, 174 untreated patients with chronic HBV infection **(B)**, and 106 NUC-treated patients with chronic HBV infection **(C)**.

**Figure 2:**
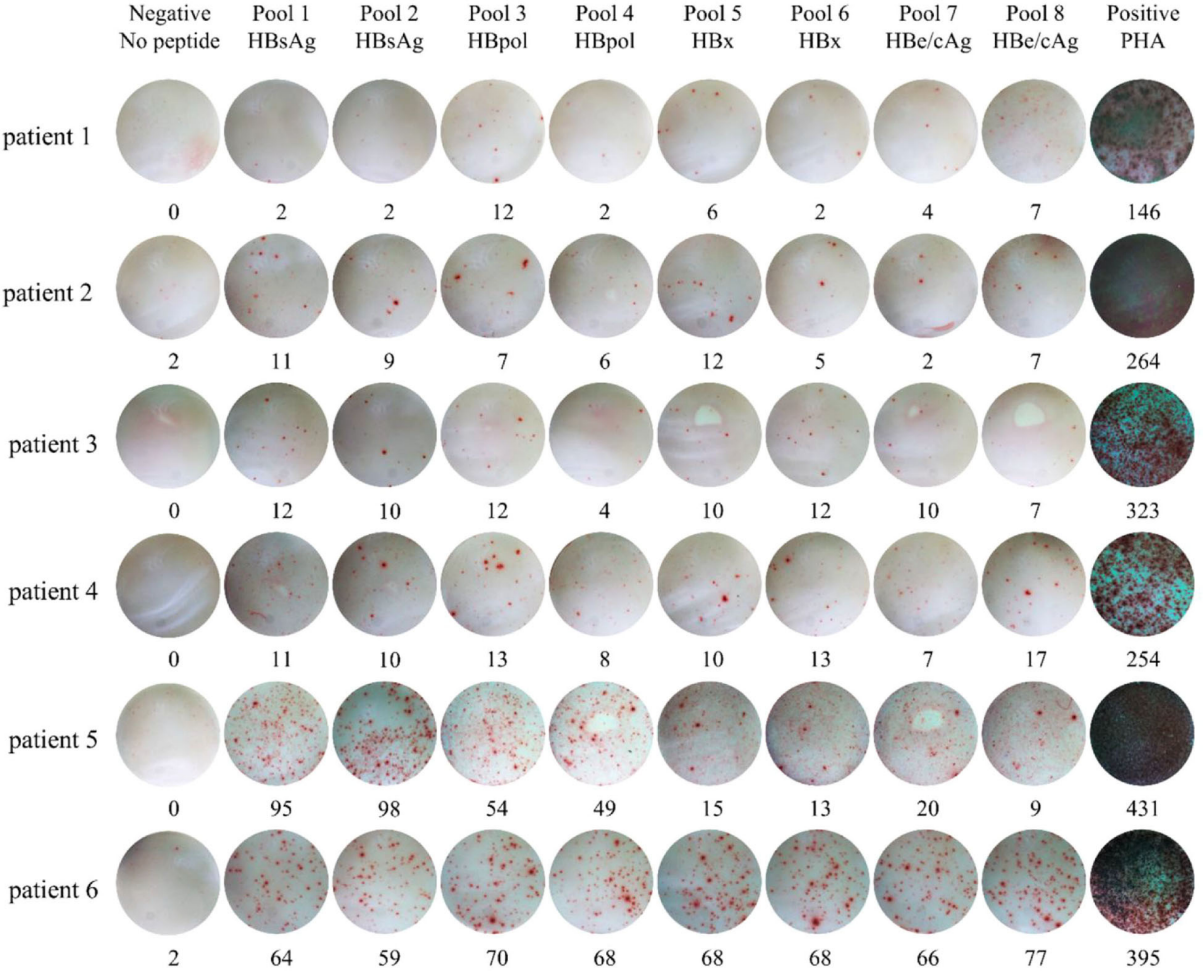
Dot plots of IFN-γ ELISpot assay for six representative pregnant patients with chronic HBV infection. PBMCs from each patient were seed into 10 wells and co-cultured for 20 hours with no peptide, eight peptide pools and PHA, respectively. Then IFN-γ was detected by ELISpot assay. PHA, Phytohemagglutinin.

A total of 283 patients with chronic HBV infection were quantified for the numbers of reactive HBV-specific T cells (SFUs) in PBMCs using the ELISpot assay. As shown in **Figure 1A**, the pregnant patients displayed significantly lower HBV-specific T cell reactivity (median 79 SFUs) than the non-pregnant patients (median 91 SFUs) (p = 0.046), but no difference to the postpartum patients (median 77.5 SFUs). Meanwhile, HBsAg- and HBpol-specific T cell reactivity (median 21 and 17 SFUs) in the pregnant group were also significantly lower than that (median 27 and 23 SFUs) in the non-pregnant group, and similar to that in the postpartum group. In the 174 untreated patients, the pregnant group displayed significantly lower HBeAg/HBcAg-specific T cell reactivity (median 13.5 SFUs) than the non-pregnant group (median 33 SFUs, p = 0.025), and the postpartum group (median 17.5 SFUs, p = 0.045) (**Figure 1B**). Notably, the 17 pregnant patients and 26 postpartum patients undergoing NUCs therapy for 3-6 months (Tenofovir Disoproxil Fumarate, TDF, 300mg QD) and the 63 non-pregnant patients undergoing NUC or pegIFN-a monotherapy or NUC/pegIFN-a combination therapy presented similar HBV-specific T cell reactivity without significant differences across the three groups (**Figure 1C**). The spot plots reactive to each peptide pool in the ELISpot assay were presented for six representative patients (**Figure 2**).

### Stratification analysis of HBV-specific T cell reactivity with clinical and sero-virological parameters

After subgrouping the 129 pregnant patients with chronic HBV infection, the ALT-normal (< 40 IU/L) patients showed significantly higher HBV-specific T cell reactivity (median 80 SFUs) than the ALT-increased (> 40 IU/L) patients (median 47 SFUs) (p = 0.021). Meanwhile, no statistical differences in HBV-specific T cell reactivity were found between subgroups with different levels of AST, viral DNA load, HBsAg and HBeAg (**Table 2**). In addition, NUCs-treated pregnant patients (TDF, 3-6 months, 300mg QD) showed similar HBV-specific T cell reactivity as the untreated pregnant patients (**Table 2**). In 58 postpartum patients (within 6 months) with chronic HBV infection, stratification analysis did not find difference in HBV-specific T cell reactivity across the subgroups, including the NUC treated or untreated subgroups (**Supplementary Table S2**). Among 96 non-pregnant women with chronic HBV infection, the patients with low HBsAg levels (<1000 IU/mL) had significantly lower HBV-specific T cell reactivity (median 71 SFUs) than the patients with moderate levels of HBsAg (1000-20000 IU/mL) (median 105 SFUs, p = 0.044), but no difference from the patients with high HBsAg level (>20000 IU/mL) (median 62 SFUs, p = 0.05) (**Supplementary Table S3**).

**Table 2:** Stratification analysis of HBV-specific T cell reactivity for 129 pregnant patients with chronic HBV infection.

Parameters	Stratification	n	HBV-specific T cells (SFUs) Median (min-max)	K-W/*P*	M-W/*P*
HBV DNA (Lg IU/ml)	< 3.0	50	77 (12 - 379)	0.828	Low vs middle 0.856
	3.0 - 5.0	27	76 (9 - 353)		Middle vs high 0.658
	> 5.0	29	78 (13 - 388)		Low vs high 0.562
HBsAg (IU/mL)	< 1000	29	97.0 (9 - 489)	0.733	Low vs middle 0.648
	1000-20000	40	75.5 (5 - 379)		Low vs high 0.383
	> 20000	34	77.5 (12 - 353)		Middle vs high 0.826
HBeAg (COI)	< 1.0	49	87.0 (9 - 489)		0.812
	> 1.0	54	82.5 (5 - 379)		
ALT (IU/L)	< 40	82	80 (9 - 489)		**0.021**
	> 40	17	47 (5 - 388)		
AST (IU/L)	< 40	90	79 (5 - 489)		0.372
	> 40	7	57 (13 - 133)		
Treatment	TDF	17	80.0 (14 - 379)		0.770
	Untreated	112	77.5 (5 - 489)		

COI, cut off index, COI = sample value/cut off value; HBeAb (reference values: 1-99 COI) COI< 1.0 means negative result. K-W, Kruskal-Wallis test; M-W, Mann-Whitney test.

### The efficacy of HBV-specific T cell reactivity to predict the hepatitis progression in pregnant patients with chronic HBV infection

**Figure 3:**
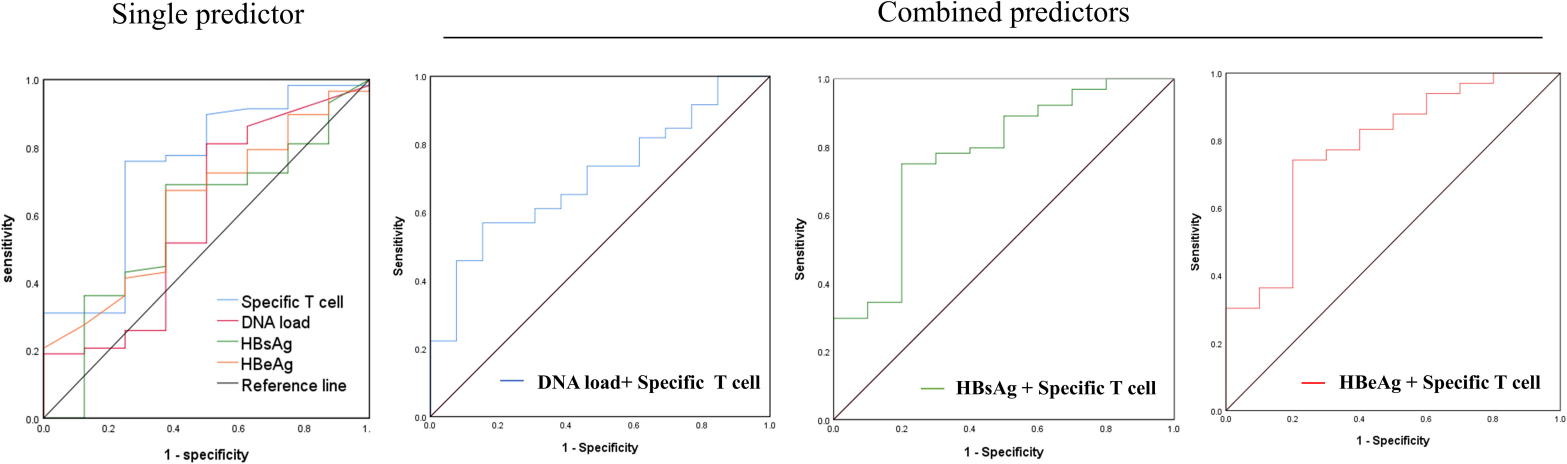
Predictive power of HBV-specific T cell reactivity on hepatitis progression in the pregnant patients with chronic HBV infection. The pregnant patients were divided into normal (ALT <40 IU/L) group and abnormal (ALT >40 IU/L) group according to the liver function at the time point of HBV-specific T cell test. ROC curve analyses of DNA load, HBsAg level, HBeAg level, HBV-specific T cells (SFUs) alone, and a combination of two factors were performed to predict hepatitis progression at the time point of HBV-specific T cell test, using R package pROC, and summarized in **Table 3**.

The predictive power of the number of reactive HBV-specific T cells in PBMCs (SFUs) on hepatitis progression was evaluated for pregnant patients. As shown in **Figure 3** and **Table 3**, HBV-specific T cells presented 0.742 AUC, 56.5 SFUs cut-off value, 75% specificity and 75.9% sensitivity for the liver function at the time point of HBV-specific T cells test, indicating that chronic HBV patients with HBV-specific T-cell reactivity less than 56.5 SFUs/4×10^5^ PBMCs were more likely to develop hepatitis (ALT> 40). Comparably, viral DNA load, HBsAg level and HBeAg level presented a predictive value lower than HBV-specific T cells. But the ROC curves for combined two factors displayed a further increase in predictive accuracy, such as AUC 0.775 for HBV-specific T cells with HBsAg level, and AUC 0.78 for HBV-specific T cells with HBeAg level.

**Table 3:** Predictive power of HBV-specific T cell reactivity on hepatitis progression in the pregnant patients with chronic HBV infection.

	Maker	Patient Number	Normal/ Abnormal	AUC	95% CI	Cut-off level	Specificity (%)	Sensitivity (%)	PPV (%)	NPV (%)	P
Single predictor	DNA load	85	72/13	0.594	0.363 - 0.825	0.1676	50.0	81.0	22.6	93.6	0.393
	HBsAg	76	66/10	0.585	0.372 - 0.798	0.0001	62.5	69.0	21.8	93.0	0.438
	HBeAg	76	66/10	0.629	0.436 - 0.823	0.0121	62.5	67.2	21.4	92.6	0.238
	Specific T cell	97	82/15	0.742	0.550 - 0.935	56.5	75	75.9	35.7	94.4	**0.027**
Combined predictors	DNA load + Specific T cell	85	72/13	0.703	0.567 - 0.839	5.57	84.6	56.9	40.7	91.3	**0.02**
	HBsAg + Specific T cell	76	66/10	0.775	0.614 - 0.936	5.71	80.0	75.0	36.2	95.4	**0.005**
	HBeAg + Specific T cell	76	66/10	0.780	0.622 - 0.939	5.87	80.0	74.2	35.9	95.3	**0.004**

ROC, receiver operating characteristic; AUC, area under the curve; PPV, positive predictive value; NPV, negative predictive value. The p values represent the significance of model.

## Discussion

Before pregnancy, during pregnancy, prenatal and postpartum, the women with chronic HBV infection need to implement comprehensive monitoring and management. Pregnancy and postpartum can induce hepatitis due to various factors, but there is no specific parameter to monitor and evaluate patient’s ability to clear virus, and to guide the appropriate time point for intervention. The number of reactive HBV-specific T cells is an precise indicator to reflect the specific cellular immune function of host antiviral infection. However, the detection of HBV-specific T cells (number or function) is much more difficult than that of antigens, antibodies and viral DNA, thus it has not been routinely carried out in clinical laboratory so far. The main limitations are as follow: 1) The HLA molecules are highly polymorphic in the population, and the antigen peptides presented by different HLA molecules have distinct sequences; 2) The T cell epitope profile in HBV antigens remains unclear and the validated epitopes are limited. In the past 34 years, only 205 CD8^+^ T cell epitope peptides and 79 CD4^+^ T cell epitope peptides have been functionally validated, and they are presented only by a few dominant HLA allotypes, thus these epitopes are unable to cover the main population in an indicated geographical region (12). Consequently, there is no universal and commercial test kit for HBV-specific T cells in clinical or research laboratories at the present.

Among the various methods to detect antigen-specific T cells, ELISpot is one of the most classical methods, which is widely used and recognized by many researchers around the world. It not only has good specificity, low reagent cost, no special instruments, easy to popularize, but also has high sensitivity in which a single cell secreting cytokine can be detected among one million cells (13). Due to the lack of broad-spectrum T-cell epitope peptide library, most of the previous clinical studies on HBV-specific T cells were tested using the overlapping peptide library (14–18) or *in silico* predicted epitope peptide (19, 20) in ELISpot assay. Unfortunately, both the overlapping and predicted peptide libraries are not real-world epitope peptides verified by cell functional experiments, and recent studies have confirmed that most of them are false epitopes (21–23).

The 103 CD8^+^ T-cell epitope peptides used in this study were functionally validated using the co-cultures of predicted candidate epitope peptides with the fresh PBMCs from more than 700 HBV-infected patients. Then, the peptide competition experiments of HLA-A molecules were further carried out using 13 engineered HMy2.CIR cell lines expressing the indicated HLA-A molecules, and followed by the molecular docking and molecular dynamics simulation experiments. The results showed that these epitope peptides can be cross-presented by 13 HLA-A allotypes. The gene frequency of each HLA-A allotype is greater than 1% in the Chinese population, and the 13 allotypes can cover more than 95% of the Chinese population. That means the in-house peptide library not only consists of real-world T-cell epitopes of HBV antigens, but also fits to the HLA polymorphism of Chinese cohort, thus can be used for random patients in ELISpot assay (10). Comparably, our data here should be more closer to the real-world functional status of HBV-specific T cells in patients than the data detected by using the overlapping and predicted peptide libraries. In addition, healthy donors were also detected using this system, but presented much higher SFUs numbers compared to the patients due to the vaccination of HBV vaccine for most Chinese individuals, so we think healthy donors cannot be used as negative control group of HBV-specific T cell detection in this study.

Previous studies in non-pregnant patients have shown that the HBV-specific CD8^+^ T cell responses are barely detectable in the peripheral blood of patients with chronic HBV infection (24), but little information is available in pregnant patients. In this study, the *ex vivo* ELISpot method was set up using an original and universal kit to quantify the number of reactive HBV-specific T cells (mainly CD8^+^ T cells), and the results show that the reactivity of HBV-specific T cells was further inhibited during pregnancy as compared with no-pregnant patients, and cannot be improved by TDF treatment. Although clinical experiences have already demonstrated that the treatment with TDF at the late pregnancy can reduce the peripheral HBV DNA load and control the hepatitis progression of pregnant patients with HBV DNA greater than 10^5^ copy/mL, but host anti-viral cellular immunity remains lower than the postpartum or no-pregnant women. The corresponding mechanisms deserve further investigation. In addition, the double-edged relationship of HBV-specific T cell reactivity to liver function remains difficult to be defined. Our data suggest that the number of reactive HBV-specific T cells, as an indicator of host adaptive immunity, is a valued predictor for hepatitis progression in pregnant patients with chronic HBV infection, especially when combined with the HBsAg level or HBeAg level. But a larger patient cohort is needed to further confirm the predictive power for the hepatitis progression 6 months or 12 months later.

Taken together, although the limitations on patient cohort and detection technique, this study truly compared the HBV-specific T cell reactivity across the pregnant, the postpartum within 6 months and non-pregnant women with chronic HBV infection, which provided preliminary observational data for further exploring the characteristics and mechanisms of specific immunity in pregnant women.

## Data Availability

The original contributions presented in the study are included in the article/**Supplementary Material**. Further inquiries can be directed to the corresponding authors.
