# Correction: Comparison of HBV-specific T cell reactivity across the pregnant, postpartum and non-pregnant women with chronic HBV infection

**DOI:** 10.3389/fimmu.2026.1792686

**Published:** 2026-02-09

**Authors:** Genju Wang, Fangping Yue, Ziyue Zhang, Yandan Wu, Ruixue Ji, Guanlun Zhou, Ying Ji, Chuanlai Shen

**Affiliations:** 1 Department of Obstetrics and Gynecology, The Second Hospital of Nanjing, Affiliated to Nanjing University of Chinese Medicine, Nanjing, Jiangsu, China; 2 Department of Microbiology and Immunology, Medical School of Southeast University, Nanjing, Jiangsu, China

**Keywords:** chronic hepatitis B infection, pregnancy, antigen-specific T cell detection, ELISpot assay, postpartum women

There was a mistake in **Figure 2** as published, which contains duplicate photographs “pools 1 and 3 for patient 1”. The corrected **Figure 2** appears below.

**Figure 2:**
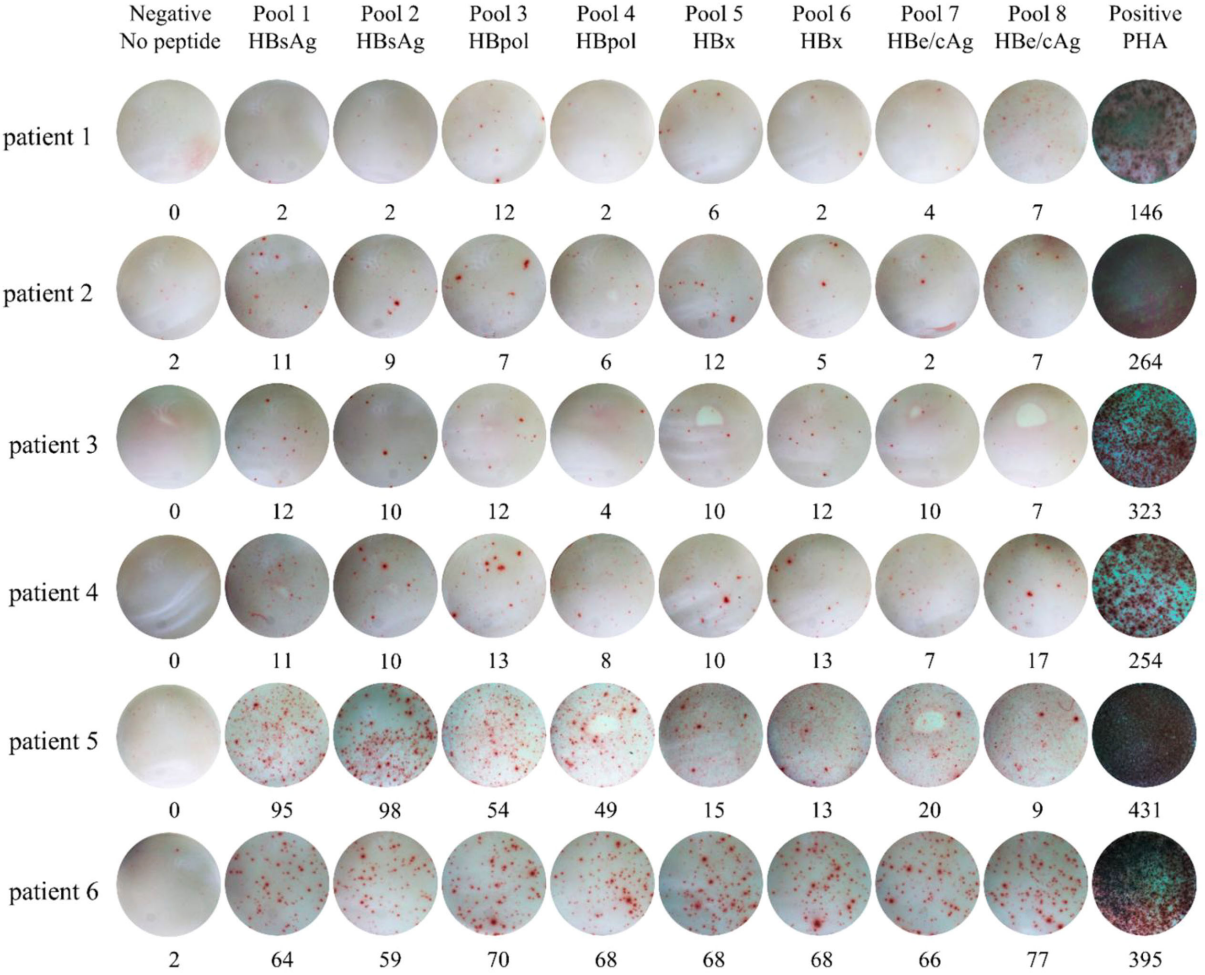
Dot plots of IFN-γ ELISpot assay for six representative pregnant patients with chronic HBV infection. PBMCs from each patient were seed into 10 wells and co-cultured for 20 hours with no peptide, eight peptide pools and PHA, respectively. Then IFN-γ was detected by ELISpot assay. PHA, Phytohemagglutinin.

The original version of this article has been updated.

Additional information was omitted and has been added in an **Acknowledgments** section, which reads as follows:

“The present study builds on our prior work (25), submitted to the Chinese Journal of Microbiology & Immunology on August 30, 2023 and published on September 30, 2024. In the latter study, conducted from September 2022 to June 2023, we tested 100 patients, including 43 pregnant women (pregnant group), 26 patients giving birth within six months (postpartum group), and 31 non-pregnant patients at childbearing age (non-pregnant group) using the in-house ELISpot assay to compare HBV-specific T cell reactivity across the three groups. That study concluded that pregnancy reduced HBV-specific T cell reactivity in the women with chronic HBV infection, but NUC treatment may have improved specific T-cell function.

The present study commenced after July, 2023, enrolling new patients to further confirm our previous findings. Finally, 283 HBV-infected patients (129 pregnant patients, 58 postpartum patients giving birth within six months, and 96 non-pregnant patients) were tested using the in-house ELISpot assay. This study was submitted to Frontiers in Immunology on July 9, 2024, and differs from our prior study in its conclusions: here, we found that while pregnancy can reduce HBV-specific T cell reactivity in women with chronic HBV infection, NUC treatment did not improve their HBV-specific T cell reactivity.

Two independent cohorts (100 patients in the first study, and 283 patients in the present study) were enrolled in these studies.”

The original version of this article has been updated.

[25] Wang G, Wu Y, Ji R, Yue F, Jiang H. Comparison of HBV-specific T cell reactivity among pregnant, postpartum and non-pregnant women at childbearing age with chronic HBV infection. *Chin J Microbiol Immunol.* (2024) 44:784–91. doi: 10.3760/cma.j.cn112309-20230830-00058.

