# Supplementary material for: Comparison of HBV-specific T cell reactivity across the pregnant, postpartum and non-pregnant women with chronic HBV infection
Source: Front Immunol. 2024 Oct 10;15:1461767. doi: 10.3389/fimmu.2024.1461767 (PMC11520174; doi:10.3389/fimmu.2024.1461767)
Supplement: Supplementary file 1 [file DataSheet1.pdf]

Comparison of HBV-specific T cell reactivity across the pregnant, postpartum and non-pregnant women with chronic HBV infection

Genju Wang, Fangping Yue, Ziyue Zhang, Yandan Wu, Ruixue Ji, Guanlun Zhou, Ying Ji, Chuanlai Shen

Table S1 Peptide pools of 103 T-cell epitopes of HBV antigens in the ELISpot assay

Peptide pool	Pool 1	Pool 2	Pool 3	Pool 4	Pool 5	Pool 6	Pool 7	Pool 8
Derived protein	HBsAg	HBsAg	HBpol	HBpol	HBx	HBx	HBeAg	HBeAg
Kinds of peptide	17	15	18	8	13	7	15	10

Table S2 Stratification analysis of HBV-specific T cell responses for 58 postpartum patients with chronic HBV infection.

**Parameters**	**Stratification**	**n**	**HBV-specific T cells (SFUs)** Median (min-max)	**K-W/ *P***	**M-W/ *P***
HBV DNA (Lg IU/ml)	< 3.0	41	89 (18 - 652)	0.436	Low vs middle 0.218
	3.0 - 5.0	4	40.5 (33 - 142)		Middle vs high 0.315
	> 5.0	7	77 (23 - 264)		Low vs high 0.954
HBsAg (IU/mL)	< 1000	4	154.5 (77 - 340)	0.083	Low vs middle 0.48
	1000 - 20000	32	113.5 (19 - 652)		Low vs high 0.068
	> 20000	15	62 (18 - 264)		Middle vs high 0.049
HBeAg (COI)	< 1.0	23	157 (19 - 492)		0.139
	> 1.0	31	81 (18 - 652)		
ALT (IU/L)	< 40	41	78 (18 - 492)		0.282
	> 40	11	98 (22 - 652)		
AST (IU/L)	< 40	47	81 (18 - 492)		0.301
	> 40	5	77 (52 - 652)		
Treatment	Treated	26	79.5 (19 - 652)		0.430
	Untreated	32	82 (14 - 492)		

Note: COI, cut off index, COI = sample value/cut off value; HBeAb (reference values: 1-99 COI) COI< 1.0 means negative result. K-W: Kruskal-Wallis test; M-W: Mann-Whitney test.

Table S3 Stratification analysis of HBV-specific T cell responses for 96 non-pregnant patients with chronic HBV infection.

**Parameters**	**Stratification**	**n**	**HBV-specific T cells (SFUs)** Median (min-max)	**K-W/ P**	**M-W/ P**
HBV DNA (Lg IU/ml)	< 3.0	64	96 (12 - 524)	0.651	Low vs middle 0.806
	3.0 - 5.0	10	85 (30 - 949)		Low vs high 0.324
	> 5.0	13	71 (13 - 536)		Low vs high 0.522
HBsAg (IU/mL)	< 1000	28	71 (12 - 363)	0.059	Low vs middle **0.044**
	1000 - 20000	46	105 (12 - 949)		Low vs high 0.558
	> 20000	12	62 (13 - 415)		Middle vs high **0.05**
HBeAg (COI)	< 1.0	51	90 (12 - 949)		0.562
	> 1.0	35	84 (12 - 524)		
ALT (IU/L)	< 40	67	85 (12 - 949)		0.205
	> 40	14	134.5 (20 - 495)		
AST (IU/L)	< 40	68	86 (12 - 949)		0.252
	> 40	13	123 (20 - 495)		
Treatment	NUCs	21	101 (12 - 495)	0.862	NUCs VS Untreated 0.929
	NUC/pegIFN	7	78 (16 - 239)		NUCs VS pegIFN 0.588
	pegIFN	35	87 (12 - 949)		Treated VS Untreated 0.775
	Untreated	33	90 (13 - 536)		NUCs + NUC/pegIFN VS pegIFN 0.750

Note: COI, cut off index, COI = sample value/cut off value; HBeAb (reference values: 1-99 COI) COI< 1.0 means negative result. K-W: Kruskal-Wallis test; M-W: Mann-Whitney test.
